# The application of explainable artificial intelligence methods to models for automatic creativity assessment

**DOI:** 10.3389/frai.2024.1310518

**Published:** 2024-10-01

**Authors:** Anastasia S. Panfilova, Ekaterina A. Valueva, Ivan Y. Ilyin

**Affiliations:** ^1^Laboratory of Psychology and Psychophisiology of Creativity, Institute of Psychology of the Russian Academy of Science, Moscow, Russia; ^2^Laboratory for the Study of Cognitive and Communicative Processes in Adolescents and Young Adults While Solving Game and Educational Problems Using Digital Environments, Moscow State University of Psychology and Education, Moscow, Russia; ^3^Department of Mathematical Theory of Intelligent Systems, Faculty of Mechanics and Mathematics, Lomonosov Moscow State University, Moscow, Russia

**Keywords:** creativity, XAI, psychological diagnostics, Urban test, CNN, transfer learning

## Abstract

**Objective:**

The study is devoted to comparing various models based on Artificial Intelligence to determine the level of creativity based on drawings performed using the Urban test, as well as analyzing the results of applying explainable artificial intelligence methods to a trained model to identify the most relevant features in drawings that influence the model’s prediction.

**Methods:**

The dataset is represented by a set of 1,823 scanned forms of drawings of participants performed according to the Urban test. The test results of each participant were assessed by an expert. Preprocessed images were used for fine-tuning pre-trained models such as MobileNet, ResNet18, AlexNet, DenseNet, ResNext, EfficientNet, ViT with additional linear layers to predict the participant’s score. Visualization of the areas that are of greatest importance from the point of view of the model was carried out using the Gradient-weighted Class Activation Mapping (Grad-CAM) method.

**Results:**

Trained models based on MobileNet showed the highest prediction accuracy rate of 76%. The results of the application of explainable artificial intelligence demonstrated areas of interest that correlated with the criteria for expert assessment according to the Urban test. Analysis of erroneous predictions of the model in terms of interpretation of areas of interest made it possible to clarify the features of the drawing on which the model relies, contrary to the expert.

**Conclusion:**

The study demonstrated the possibility of using neural network methods for automated diagnosis of the level of creativity according to the Urban test based on the respondents’ drawings. The application of explainable artificial intelligence methods to the trained model demonstrated the compliance of the identified activation zones with the rules of expert assessment according to the Urban test.

## 1 Introduction

### 1.1 The problem of diagnosing creativity using drawing tests

Creativity assessment is the process of evaluating an individual’s creative thinking abilities and capacity to generate novel and valuable ideas.

The first test for creativity is often attributed to J.P. Guilford, who developed the cubic model of the structure of intelligence in the 1950s. Divergent thinking, which Guilford assumed to be the essence of creative thinking, was one of the components in this model. Divergent thinking is the process of generating diverse ideas, as opposed to seeking a single correct solution. Divergent thinking tests involve tasks where individuals are asked to come up with as many solutions as possible in response to a given problem. For example, tasks may involve generating as many items as possible that meet a set of specific criteria, finding similarities among dissimilar objects, identifying novel uses for an object, coming up with as many consequences of a hypothetical situation as possible, and so on.

The widely known and extensively psychometrically validated test for divergent thinking is the Torrance Test of Creative Thinking. It was developed by E. Paul Torrance in 1966 and revised four times (in 1974, 1984, 1990, and 1998) and was translated into 35 languages worldwide (Kim, 2006). The test has verbal and non-verbal versions. The non-verbal part of the test has gained the most popularity due to its simplicity, speed of administration, and minimal adaptation effort for non-English-speaking countries. The test results can yield an overall creativity index, consisting of five norm-referenced scores (originality, elaboration, fluency, abstractness of titles, and resistance to premature closure).

K. Urban and G. Jellen criticized the idea that creative thinking was only associated with divergent skills and the speed of generating ideas. Instead, they introduced a fresh approach to assess creative abilities (Urban, 2004). Urban’s theoretical concepts are gestalt-oriented and are based on a componential model of creativity, which includes both cognitive factors (divergent thinking, general thinking ability, knowledge base, specific knowledge and skills in a particular area) and personality factors (cognitive motivation, perseverance, concentration, openness to experience, and tolerance for ambiguity). Their Test of Creative Thinking-Drawing Production (TCT-DP) was specifically designed to assess not only quantitative but also qualitative aspects of creative output, such as content, composition, elaboration, completeness, risk propensity, nonconformity, humor, etc. Additionally, when developing their test, the authors aimed to make it: (1) applicable to different age groups; (2) simple and cost-effective in administration, results processing, and interpretation; (3) culturally independent.

Some researchers highlight the importance of incorporating creativity into gifted identification procedures—it allows for a more comprehensive evaluation of students’ cognitive abilities (Luria et al., 2016; Desmet et al., 2021). But for most creativity measures scoring procedures could be challenging due to several reasons. For example, scoring relies on human judgment, which can introduce subjectivity and variability among scorers. Creative responses may be ambiguous or unconventional, making it difficult to determine their appropriateness or quality. Sometimes it is problematic to establish clear and consistent scoring criteria. Finally, scoring is time-and resources-consuming, especially if one needs to evaluate several hundreds of protocols.

In this study, we applied explainable artificial intelligence methods to models for the automatic assessment of TCT-DP results. This test has a wide range of scores (from 0 to 72) and a set of 14 clear evaluation criteria. These allow us to test our models at different creativity levels and to compare model’s activation zones with expert ratings.

### 1.2 Automated creativity diagnostics

Historically, the first studies on automatic evaluation of creativity tests focused on verbal tests, particularly those assessing divergent thinking, such as the Alternate Uses Test (Valueva et al., 2024). Typically, expert evaluations are used for such tests. The paradox in automatic creativity diagnostics is that while researchers criticize the subjective nature of expert opinions, they are compelled to compare machine learning assessments with these same expert evaluations. Thus, the effectiveness and accuracy of these automated systems are measured against the very expert evaluations they aim to replace or supplement. In practice the unreliability of expert evaluations is often overlooked, and these evaluations serve as benchmarks for model quality. The best models are those that most closely replicate human assessments.

In contrast to the assessment of verbal creativity, there is less research on automating the diagnosis of creativity through drawing tests. Drawing tests generally have clearer systems for evaluating originality, such as the stereotypical use of stimulus elements, the diversity and number of details etc., which are quantifiable. For example, Urban’s TCT-DP provides a clear quantitative evaluation system where the only subjective criterion is “Humor,” contributing minimally to the overall score (about 8%, with a maximum score of 6 out of 72).

Noteworthy is the work of (Cropley and Marrone, 2021), which presents a model for automatically assessing the results of the Urban drawing test using convolutional neural networks. The study used a total of 414 images to solve the problem of classifying the result into 3 levels. The accuracy of the model based on fine-tuning MobileNet V2 (Sandler et al., 2018) by adding a classification layer for high, medium and low levels was 94.2%. Table 1 contains the results of training models using the TU-Berlin sketch dataset (Deimel and Brock, 2016) and images from Urban test. The distribution of elements across samples (train, test and validation) differs in different studies, but is the same within the one study. The more effectively a pre-trained model can classify hand-drawn images, the more effectively it can learn for the task of assessing creativity in drawing tests. As a result, AlexNet (Krizhevsky et al., 2012) and GoogleNet (Shelhamer et al., 2014) demonstrated similar results of about 75% accuracy. In the work of Eyiokur et al. (2018) the author presented the results of additional training of VGG16 (Simonyan and Zisserman, 2014) with an accuracy of 79.72%. The work of Liang et al. (2022) currently demonstrates the highest efficiency of fine-tuning ResNet50 (He et al., 2015) pre-trained model with a classification accuracy of 86.6%.

**Table 1:** The comparison classification results on TU-Berlin sketch dataset and Urban test images.

Model	Paper	Dataset	Accuracy
AlexNet	Zhang et al. (2016)	TU-Berlin	75.02%
GoogleNet			75.25%
VGGNet			76.53%
VGG16	Eyiokur et al. (2018)	TU-Berlin	79.72%
ResNet50	Liang et al. (2022)	TU-Berlin	86.6%
MobileNet V2	Cropley and Marrone (2021)	414 images (Urban test)	94.2%

### 1.3 Methods of explainable artificial intelligence in computer vision tasks

The use of CNN (convolutional neural networks) is one of the most effective approaches in image recognition and segmentation problems. Models that solve these problems contain a set of convolutional layers, pooling layers, and are completed with a number of fully connected layers for transforming data into estimates. Existing approaches to understanding the operational mechanism of trained models with a similar structure are divided into 2 categories: (1) explanation of the model’s operation through modification of the input image and analysis of the influence degree on the output result to determine the most relevant parts of the input image; (2) methods that attempt to analyze the operation of nested layers in general rather than in particular (Arrieta et al., 2019). The work of Bach et al. (2015) presents the Layer-wise Relevance Propagation approach, which allows one to evaluate the influence of each pixel on the model output and build a heatmap that is convenient for human interpretation.

Subsequent developments in visualization methods led to the creation of Grad-CAM (Gradient-weighted Class Activation Mapping) (Selvaraju et al., 2016), which uses data on the gradients of the final convolutional layer, highlighting the corresponding regions. The second category of approaches is presented as an example in the work of Mahendran and Vedaldi (2014), in which the authors proposed a general method to invert representations, their method uses only information from the image representation and a generic natural image prior, starting from random noise as initial solution, and hence captures only the information contained in the representation itself. Also, the Deep Generator Network (Nguyen et al., 2016) is included in approach 2 and it generates the most representative image for the output layers.

Explainable artificial intelligence (XAI) methods are designed to increase confidence in the technologies, which are developed based on Artificial Intelligence, due to the opportunity to analyze a set of features that affect the model’s prediction and identify artifacts that negatively affect its operation.

## 2 Materials and methods

### 2.1 Urban test

As a part of the test, the participant is asked to complete an unfinished free-form drawing using a simple pencil without using a ruler, eraser or other improvised means. The test form shows six objects—a dot, a semicircle, a wavy line, a right angle, a dotted line, enclosed in a large frame, and an unfinished square located outside the frame. There are 2 forms of the test—A and B. Form B is an image of Form A flipped 180 degrees. According to the test manual, the drawings are scored and interpreted across 14 categories (Urban and Jellen, 1996; Urban, 2004):

1. Continuations (Cn): Any use, continuation or extension of the six given figural fragments.
2. Completion (Cm): Any additions, completions, complements, supplements made to the used, continued or extended figural fragments.
3. New elements (Ne): Any new figure, symbol or element.
4. Connections made with a line (Cl) between one figural fragment or figure or another.
5. Connections made to produce a theme (Cth): Any figure contributing to a compositional theme or “gestalt.”
6. Boundary breaking that is fragment dependent (Bfd): Any use, continuation or extension of the “small open square” located outside the square frame.
7. Boundary breaking that is fragment independent (Bfi).
8. Perspective (Pe): Any breaking away from two-dimensionality.
9. Humour and affectivity (Hu): Any drawing which elicits a humorous response, shows affection, emotion, or strong expressive power.
10. Unconventionality, a (Uc, a): Any manipulation of the material.
11. Unconventionality, b (Uc, b): Any surrealistic, fictional and/or abstract elements or drawings.
12. Unconventionality, c (Uc, c): Any usage of symbols or signs.
13. Unconventionality, d (Uc, d): Unconventional use of given fragments.
14. Speed (Sp): additional points may be awarded for speed if the total score for other criteria exceeds a certain threshold.

Each scale is scored in a range of either 0 to 6 points or 0 to 3 points. According to the manual for the test methodology, it is customary to evaluate only the overall indicator of the Urban test (the sum of points), because the test is designed to determine the overall level of creativity.

### 2.2 Study design and population

The study involved 1823 participants aged from 6 to 45 years (Mean_age_ = 15.9, SD_age_ = 3.6; 39% of men). The sample includes students of secondary schools and students of higher educational institutions. Data collection was carried out over several years as a part of different research projects. As part of Project 1 in 2007, results were obtained from 184 schoolchildren aged 14 to 18. The assessment of creativity was conducted as part of a collaborative Russian-German study (Valueva and Ushakov, 2010). In Project 2 in 2009, data were collected from 132 schoolchildren aged 6 to 10 as part of a psychological assessment at school. During Project 3, which ran from 2009 to 2011, 985 schoolchildren aged 13 to 17 were tested. The assessment of creativity was part of an educational project aimed at developing students’ emotional abilities (Valueva et al., 2018). In Projects 4, 5, and 6, conducted in 2008–2009, 2010–2011, and 2012 respectively, data were collected from 521 students of various specialties aged 17 to 45. The testing for the last three projects was carried out as part of the “Psychology of Creativity” and “General Psychology” courses within the university’s educational programs.

Respondents received a paper Urban test form on which they indicated their data and had to complete the task. The form also indicated the time it took the participant to complete the task. Although some participants completed both Form A and Form B, the analysis was performed on Form A only.

**Figure 1:**
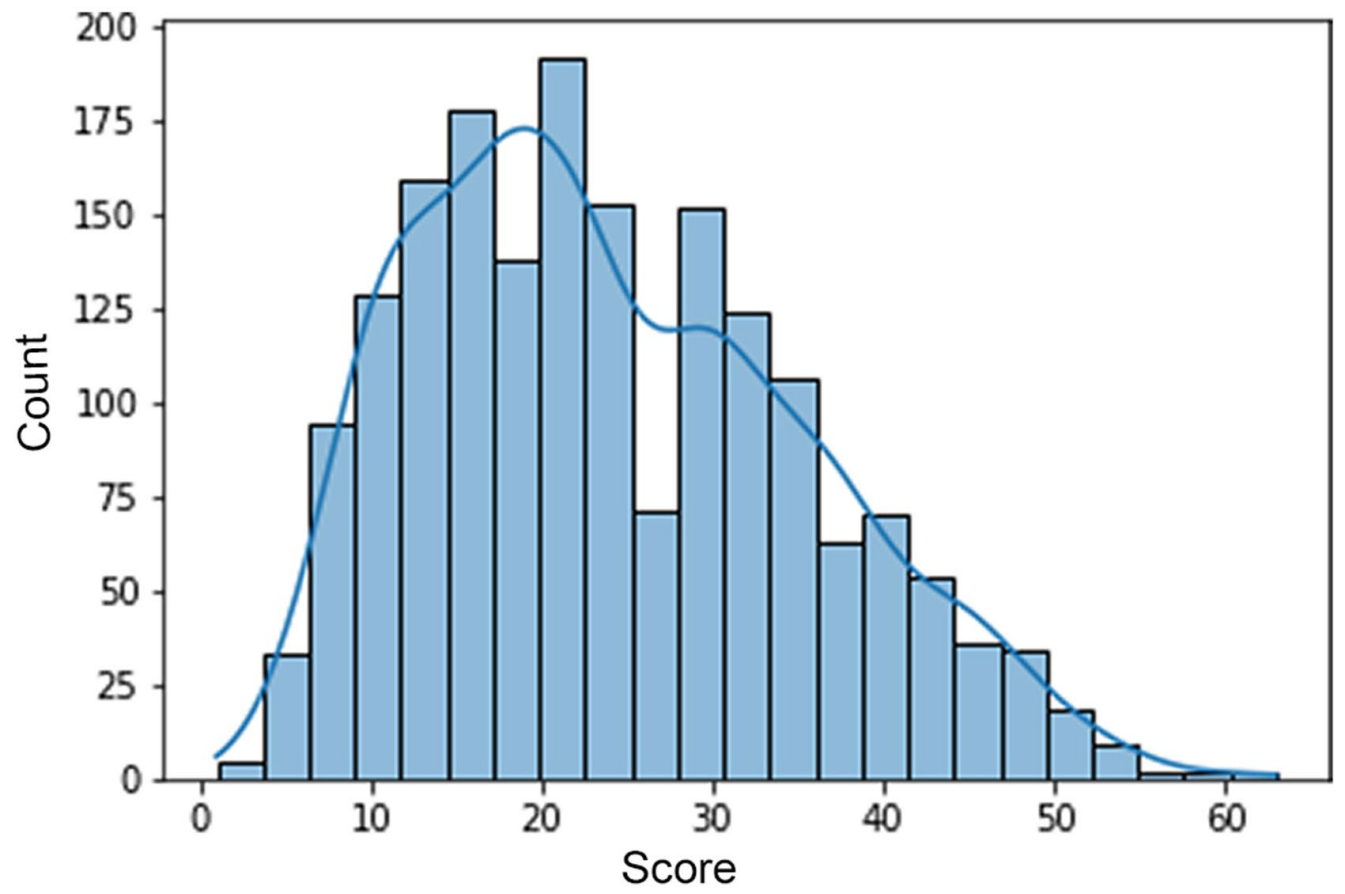
Distribution of the Urban test results.

The forms were evaluated by experts in accordance with the instructions and the data were entered into a table. Next, the paper forms were scanned with a resolution of 3507x2481px and 300 dpi. However, due to the fact that the upper part of the form contained the data of the test taker, all drawings were trimmed from the top up to the size of 3200px. A histogram of the distribution of test participants’ final scores on the test is shown in Figure 1 and ranged from 1 to 63.

### 2.3 Model development

One of the common techniques for applying deep learning to specific tasks using only a small datasets available for that task is fine-tuning. The CNNs are first pre-trained on the large general datasets (like ImageNet), which allows pre-tuning of the model weights on a vast dataset. Then, the models are adapted by partial training of the last layers on the task-specific smaller dataset. Usually only a fraction of model layers is tuned in the fine-tuning to avoid overfitting on a smaller dataset. This work considers seven pre-trained models: MobileNet V2 (Sandler et al., 2018; Mobilenet_v2, n.d.), AlexNet (Krizhevsky et al., 2012; AlexNet, n.d.), ResNet18 (He et al., 2015; Resnet18, n.d.), ViT (Dosovitskiy et al., 2020; Hugging Face, 2024a), EfficientNet V2 (Tan and Le, 2021; Hugging Face, 2024c), ResNext101 (Xie et al., 2016; Hugging Face, 2024d), DenseNet121 (Huang et al., 2016; Hugging Face, 2024b). The EfficientNet and Vit models were pre-trained on the ImageNet-21 k dataset, the remaining models were pretrained on ImageNet-1 k. ResNext101 was additionally trained on the Instagram-1B hashtags dataset. The PyTorch and Timm libraries was used to implement the learning process.

#### 2.3.1 Image preprocessing

During the experiments, the optimal image size of 1240x1600px was selected.

Also, for all models the following image transformations for data augmentation were carried out.

- random horizontal flip;
- random vertical flip;
- random rotation with 55 degrees;
- color jitter: randomly change the brightness, contrast and hue;
- random adjust sharpness with factor 2.

Each tensor containing drawing data was normalized with the following parameters: mean [0.485, 0.456, 0.406] and standard deviation [0.229, 0.224, 0.225]. We used transforms method from torhvision library (Torchvision, n.d.).

#### 2.3.2 Fine-tuning pre-train models

The training procedure for the models described below included updating the weights of all layers of the modified structure of the pre-trained model, which assumes a small number of epochs and a low learning rate. In the pre-trained models, we replaced the block of “classifier” layers with our own sequence of layers. The respondent’s test score is assumed as the output of the model, that is, the regression problem has been solved.

The “classifier” block of the AlexNet model was replaced with a sequence of fully connected layers with features size: 1000, 300, 1. Also, these layers alternate with ReLU and Dropout (0.25) layers. The “classifier” block of the EfficientNet V2, MobileNet V2, DenseNet121 models was replaced with a sequence of Dropout (0.25) layer and fully connected layer. The output layer of the ResNet18 and ResNext101 was modified to output dimension equal to 1. In the pre-trained model based on the ViT architecture, a “pooler output” is used, which is connected to fully connected layer with output dimension equal to 1.

The hyperparameters for the proposed model used during training is shown in Table 2.

**Table 2:** Hyperparameters for the proposed models.

Hyperparameters	Name/value
Rate of learning	3e-05
Batch size	4
Loss function	L1 (mean absolute error)
Optimizer	Adam
Beta1	0.9
Beta2	0.999

To assess model accuracy, the R^2^ (coefficient of determination) regression score function metric was used, and the target variable was normalized using the following formula Equation 1:

(1) $$y_{i}=\frac{y_{i}-\min\left(y\right)}{\max\left(y\right)-\min\left(y\right)}$$

The models were trained by dividing the sample into training, validation and testing in a ratio of 70, 15, 15%.

### 2.4 Explainable artificial intelligence method

The chosen Grad-CAM method is the most balanced in terms of the required computing resources, implemented in a number of libraries, however, the most suitable if take development tools is PyTorch Grad-CAM (Gildenblat et al., 2021). The method is based on the idea that fully connected layers lose spatial information, which is preserved in successive convolutional layers. Thus, it can be assumed that the last convolutional layers of the model contain high-level data, for example, specific to a particular class, but at the same time preserve spatial information.

A topical issue is to determine the layer to which it is planned to apply the Grad-CAM method within a specific model. Thus, for the ResNet18 model it is recommended (Gildenblat et al., 2021) to use the last layer in the layer4 block. For AlexNet, this could be the last convolutional layer (number 10) of the model. In the MobileNet model, this is the last activation layer (number 18).

Most XAI libraries in this area are focused on image classification, however, this library allows you to analyze activation zones in regression problems.

## 3 Results

### 3.1 Models performance results

**Figure 2:**
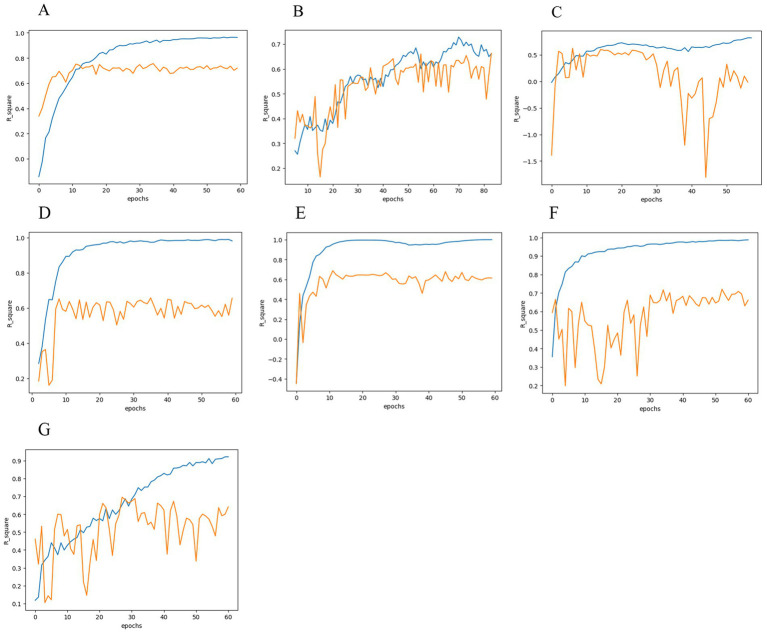
Models accuracy. **(A)** MobileNet V2. **(B)** AlexNet. **(C)** ResNet18. **(D)** ViT. **(E)** DenseNet121. **(F)** ResNext101. **(G)** EfficientNet V2.

Table 3 contains the training results of 7 models (MobileNet V2, AlexNet, ResNet18, DenseNet121, ViT, ResNext101, EfficientNet V2). Model training graphs are presented in Figures 2A–G demonstrate that a given number of epochs is sufficient to achieve model overfitting and makes it possible to identify a better fit of the model’s prediction to the ground truth. The MobileNet-based model shows the best prediction accuracy result, which can be attributed to a more optimal architecture.

**Table 3:** Models evaluation.

Model	R^2^ (validation)	R^2^ (test)
ResNet18	0.62	0.58
MobileNet V2	0.76	0.76
AlexNet	0.66	0.65
ViT	0.66	0.72
DenseNet121	0.67	0.67
EfficientNet V2	0.7	0.68
ResNext101	0.72	0.69

### 3.2 Explainable artificial intelligence results

**Figure 3:**
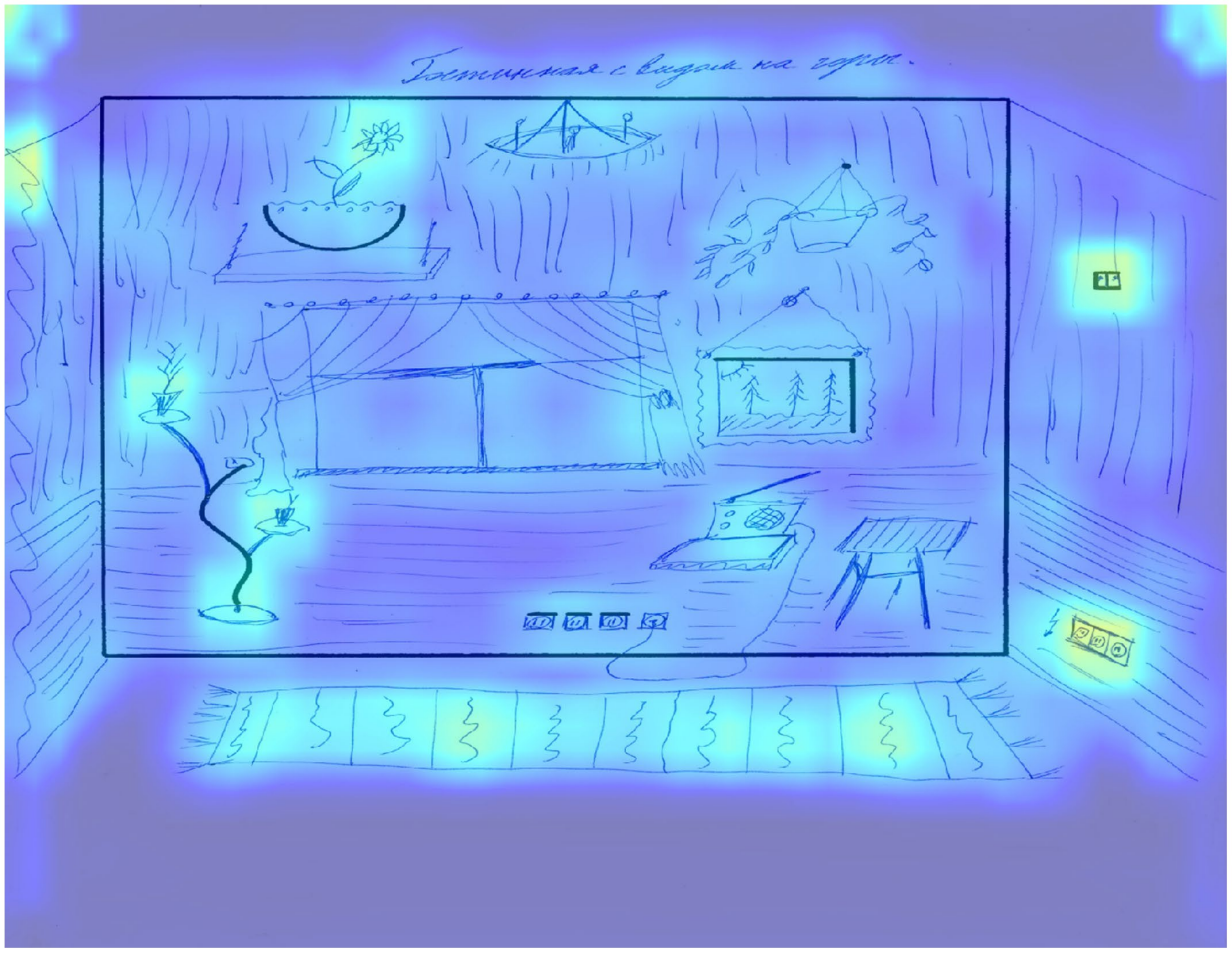
The result of applying Grad-CAM to a trained model for a drawing of a participant with a high level of creativity.

Applying the Grad-CAM method allows for the visualization of regions in the drawings that significantly contributed to the model’s predictions. We propose to examine low, medium, and high creativity drawings from the test set to correlate expert scores with the areas that show the greatest influence on the model’s predictions. Figure 3 shows an example of a image that scored 52 points according to an expert assessment and the same score according to the model.

This work received the maximum score for the first 8 criteria: continuations, completion, new elements, connections made with a line between one figural fragment or figure or another, connections made to produce a theme, boundary breaking that is fragment dependent, boundary breaking that is fragment independent, perspective, as well as 3 points for the unconventional use of given fragments criterion and 1 point for the speed criterion. In terms of activation zones, areas that extend beyond the border of the picture, especially the carpet below, are highlighted, which creates perspective, as well as the image of specific objects, for example, a socket. The unfinished square on the right side, which the respondent entered into the drawing, is highlighted separately, for which a separate criterion number 6 is presented. Next, it should be noted that if this element is not used in the drawing, it is not highlighted as a significant area. The flower at the top of the picture also stands out, since in this area respondents mostly do not depict anything, using this element as the sun. In this work the representation of this element as a flower pot can be seen as well as a new use of the space above it.

The two flowers located in the lower left corner, for which the basic element is represented by a wavy line, also represent an example of a non-trivial use of an element that often turns into a tree, which made it possible to create new elements by linking them with the basic one. The use of a drawing area free from basic elements is determined by the new elements criterion. The name of the drawing, which the respondent came up with, is also highlighted as a significant area and influences the expert assessment according to criterion 5, which contributes to the formation of the theme of the drawing, while its content is insignificant.

**Figure 4:**
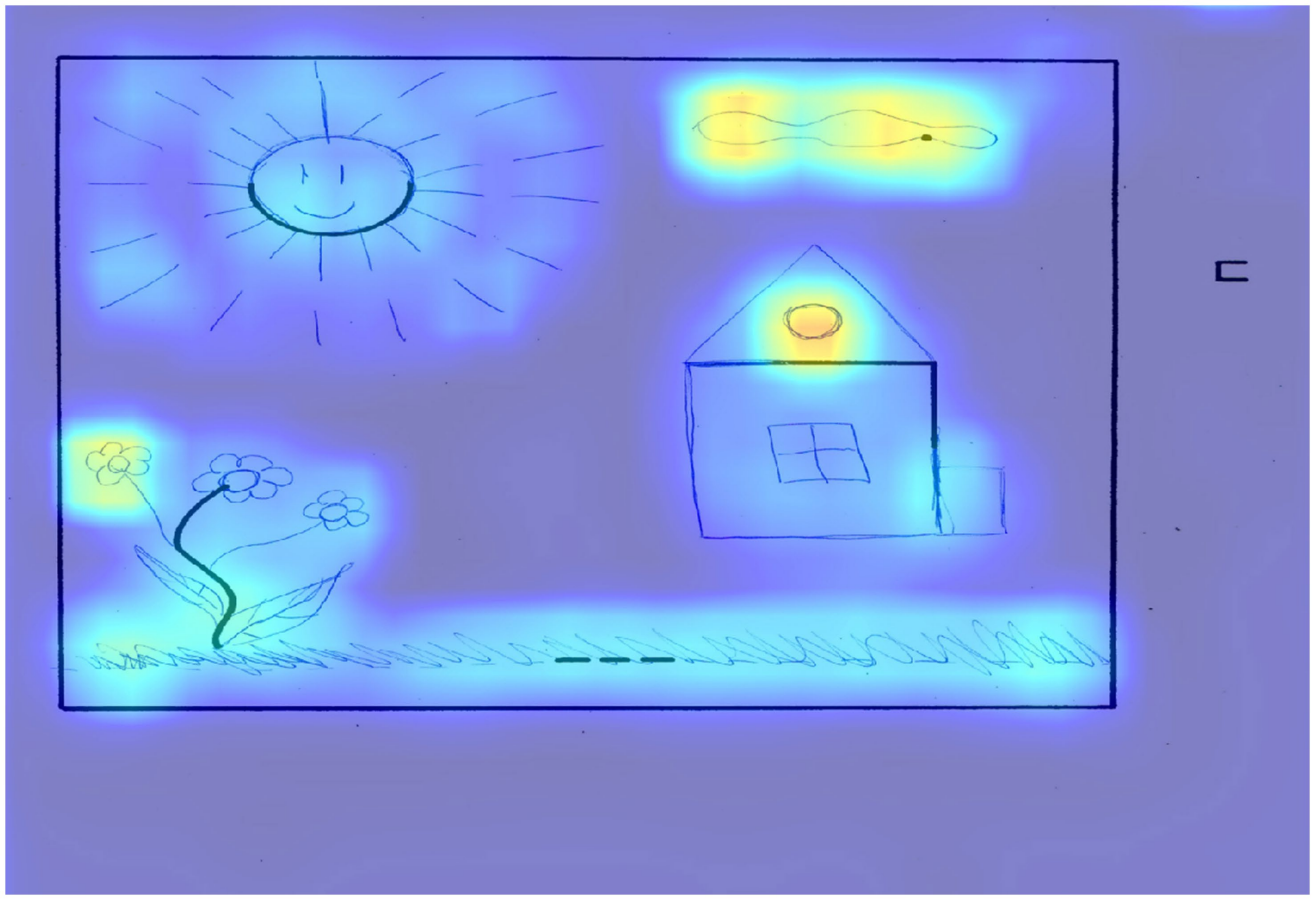
The result of applying Grad-CAM to the trained model for the drawing of a participant with a low level of creativity.

Figure 4 shows an example of a drawing from a participant with a low level of creativity.

This figure shows a typical plot that received 17 points according to an expert assessment, and 19 points according to the model prediction. According to an expert assessment, this work received 5 points according to the criteria of continuations and completion, since the square on the right side of the figure was not completed. In the figure, the selection of areas around the five elements that were supplemented and the absence of selection of the square can be observed, since it was not supplemented. The expert scored 6 points for the connection made to produce a theme criterion, which included all elements that contributed to the theme of the drawing.

In terms of significant areas, we can note the cloud and the house window above, which have the greatest significance for the model in this figure. The expert also noted 1 point for the criterion connections made with a line between one figural fragment or figure or another, which, from the point of view of activation zones, can be interpreted as grass that connects a dotted line with a wavy line, complemented like a flower.

**Figure 5:**
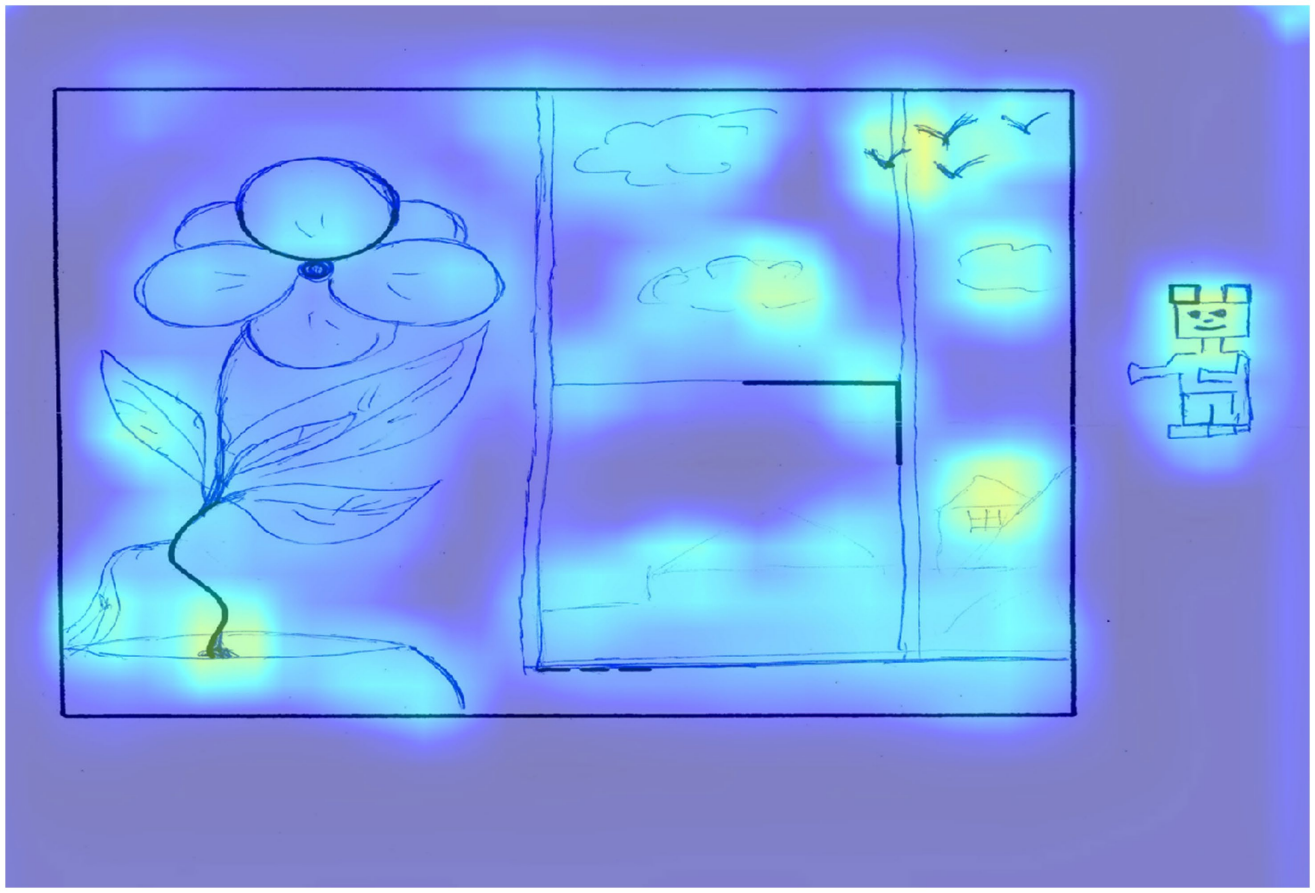
The result of applying Grad-CAM to the trained model for a drawing by a participant with an average level of creativity.

Figure 5 depicts an example of a drawing with an average level of creativity, which received 35 points according to the expert’s assessment and the model’s prediction.

The expert scored 6 points each for the following criteria: continuations, completion, connections made with a line between one figural fragment or figure or another, connections made to produce a theme, boundary breaking that is fragment dependent. In terms of the most significant areas from the XAI point of view, one can note the inclusion of all elements in the drawing, including the square on the right side of the figure. The connection of different figures by lines is also expressed quite clearly, for example, the flower on the left is completely highlighted as a single fragment due to the fact that there are connecting lines; the corner and three lines are similarly connected.

According to the new elements criterion, the expert gave 3 points, since there were two categories of such elements: clouds (3 pieces) and house. Another interesting point is that all the elements are also highlighted in the drawing. According to the perspective criterion, the expert gave 2 points, since the perspective in the window and in the flower pot might be observed, which was also highlighted by the algorithm. It must be noted that the algorithm practically does not highlight the flower and the semicircle with which it is formed, which may be due to the typical use of this element.

Based on the analysis of the figure, it can be concluded that the regions highlighted by the model often correlate with scores according to expert assessment criteria. This does not necessarily mean that the model has “learned” the criteria, but it indicates that the presence of unique elements in specific areas of the drawing, as well as a sufficient area of interconnected elements, contributes to higher scores. Additionally, it is noted that bright areas frequently appear in the upper corners of the drawings, which may be due to the operation of the Grad-CAM algorithm.

**Figure 6:**
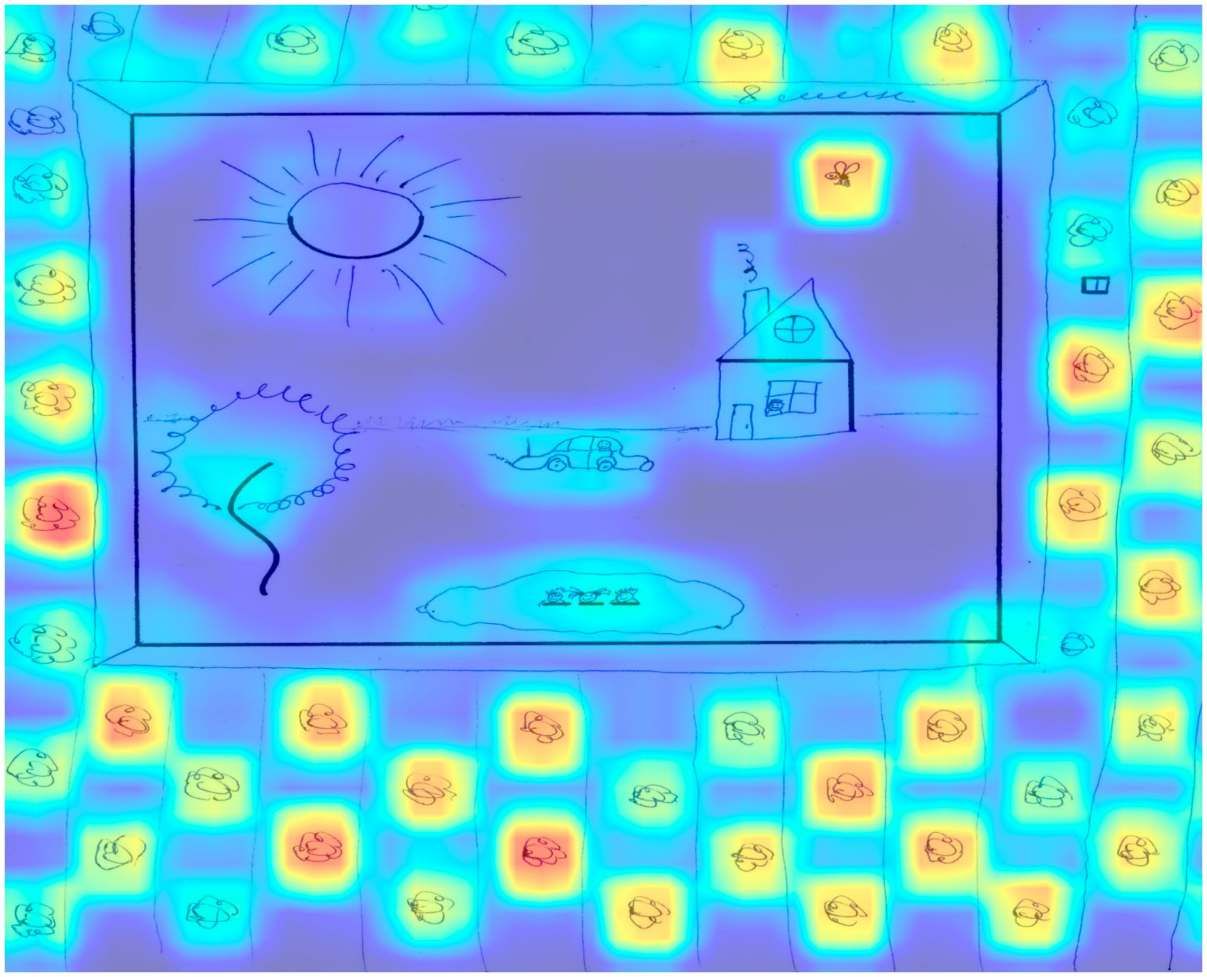
The result of applying Grad-CAM to the trained model for a drawing by a participant, when model overestimated creativity scores.

Let us analyze the cases where the expert’s ratings does not match the model’s predictions. Figure 6 present examples where the model overestimated creativity scores. The expert’s evaluation of drawing 6 is lower (38) than the model’s prediction (68). The difference is huge and, judging by the highlighted areas, is due to a large number of elements drawn outside the frame. However, all these elements are identical, so the expert, according to the guidelines, only gives 2 points for this (“If elements and additions are repeated in the same or very similar style, a maximum of only two points is awarded”). Therefore, it turns out that the model overestimates creativity when faced with high productivity (high space filling), while the expert evaluates not only the quantity but also the content of drawings.

**Figure 7:**
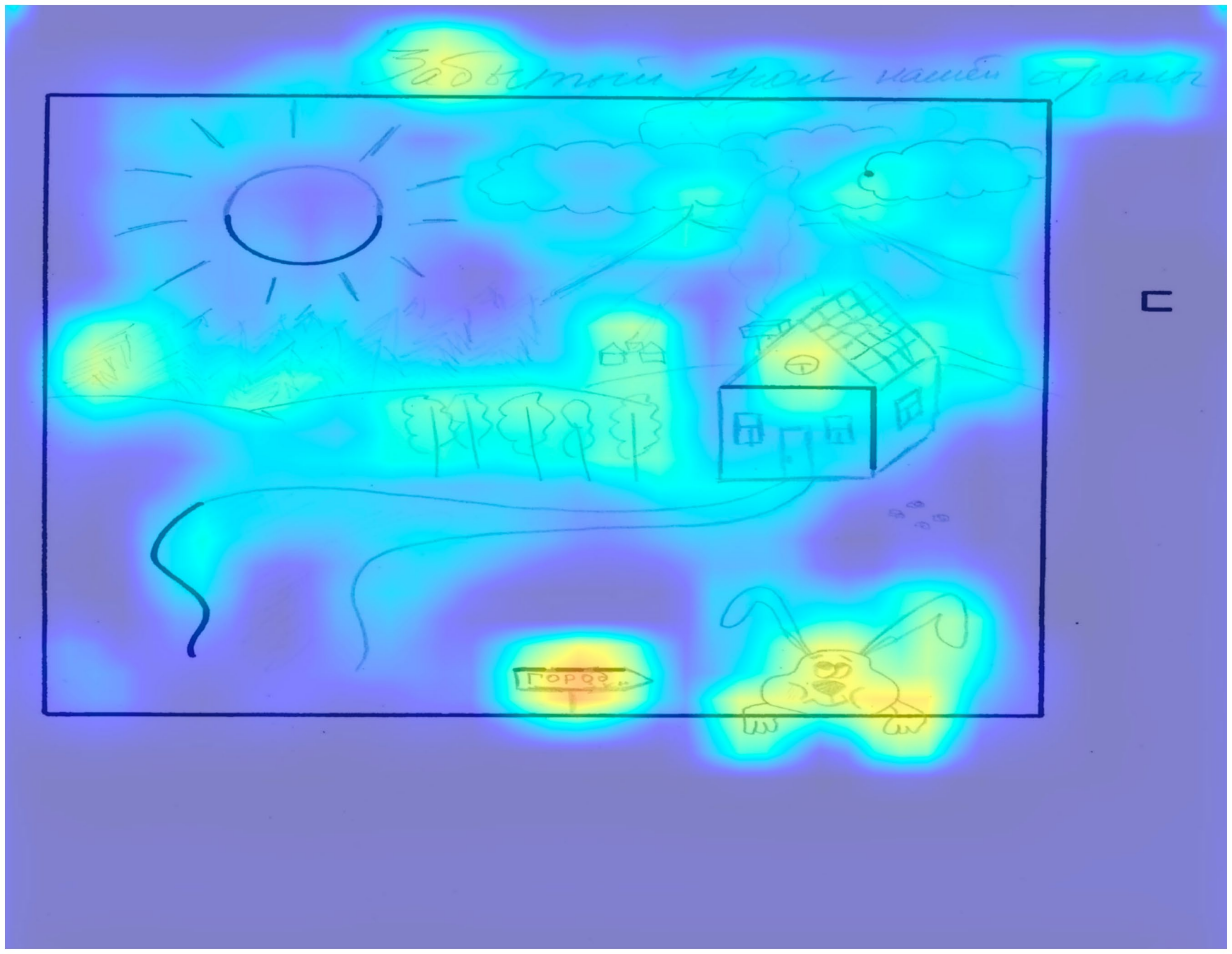
The result of applying Grad-CAM to the trained model for a drawing by a participant, when model underestimated creativity scores.

Now let us move on to the opposite cases – cases of the model underestimating the creativity scores (Figure 7). The expert’s evaluation of the drawing in Figure 7 is higher (46) than the model’s prediction (33). The difference is 13 points. This difference can be explained by the following components of the expert assessment that the model does not capture. Firstly, it is the affective component. According to the guidelines, points in the “Humor” (Hu) category are given to “drawings that demonstrate strong affective involvement or emotional mood.” In this case, the title of the drawing “Forgotten Corner of Our Country” was perceived by the expert as emotional involvement (nostalgic feelings), which was rated at 3 points. Next, the model does not consider the speed parameter, for which additional points are also awarded (in this case, the drawing was completed in 6 min, giving an additional 3 points). Another parameter likely underestimated by the model is line connections (Cl). According to the guidelines, “Each drawn connection between two elements… is rated at 1 point.” This drawing contains many drawn connections, which earned it the maximum 6 points. Thus, 12 out of 13 points can be explained by the model’s imperfection in registering three parameters.

Analysis of the model’s erroneous predictions indicates the following issues. Firstly, the model’s inability to substantively analyze the content of the images leads to quantity being mistaken for quality. Overall, such a system works fine because there is a correlation between the overall score and the number of drawn elements. However, this strategy sometimes leads to the overestimation of drawings characterized by high space filling with stereotypical elements. Secondly, there are problems with assessing subjective criteria such as humor, emotional involvement, and thematic content. The speed parameter is objective and can be included in the analysis in further studies.

## 4 Discussion

The current study shows that the most suitable pre-trained model among those considered is MobileNet, which demonstrates an accuracy of predicting the overall creativity score of 0.76. However, the creation of such diagnostic models is associated with the problem of trust in the results of their work and understanding the details of the picture that the model is guided by when constructing a forecast. To overcome this problem, we applied one of the Grad-CAM methods of explainable artificial intelligence to the test sample, which made it possible to correlate the areas identified by the algorithm with the expert assessment scores according to the criteria and qualitative analysis of the results.

A qualitative analysis of the activation zones of all drawing s of the test sample allows to draw a number of conclusions:

1. The model takes into account the continuations criterion, without highlighting the basic elements if they are not included in the figure.
2. The completion criterion from the model point of view is interpreted as elements added to the areas of the basic figures at some distance from them.
3. The new elements criterion is seen as the model’s selection of objects located in areas of the drawing, that are not related to the basic shapes (for example: the upper right corner).
4. The criteria connections made with a line between one figural fragment or figure or another and connections made to produce a theme are expressed in the combination of fragments of the drawing into a single activation area due to the connection of figures through various elements (line, shading).
5. Boundary breaking that is fragment dependent: Any use, continuation or extension of the “small open square” located outside the square frame is diagnosed in the best way, as this figure is not used in the drawing, this area never falls into the activation zone.
6. The criterion of boundary breaking that is fragment independent can be expressed in a drawing as the highlighting of lines extending beyond the square by an algorithm, creating a perspective, however, these features can also be attributed to the selection of signatures and additional elements outside the square according to the criterion of unconventionality (any usage of symbols or signs).

The remaining criteria with this approach cannot find their expression in the results of applying XAI methods. However, we consider the result obtained to be quite progressive. It can be pointed out that the clarity of the formulation of the criterion affects the result of the expert’s work, and, accordingly, the model. Thus, the criterion for using “small open square” is quite clearly manifested in the corresponding activation zones, in contrast to humor and affectivity.

For future studies, it can be recommended to involve a number of experts for assessment, as well as the use of image segmentation by an expert according to various criteria. Isolating significant areas of the image in accordance with the criteria can make it possible to correlate these areas with the zones selected by the algorithm. The coincidence of areas can be assessed using Dice or Jaccard coefficient etc. (Kofler et al., 2021). It is also possible to train an image segmentation model, which, by no means, involves the problem of manually labeling images of the training set within the framework of various expert assessment criteria.

The most promising direction seems to be the use of a transformer-based model, trained on hand drawings, which can then be further trained on our sample. At the moment, there are a number of datasets of similar images, for example: QuickDraw (GitHub, n.d.) or TU-Berlin, on the basis of which image classification models can be trained, which is later to be adapted to the current task using transfer learning, which will certainly increase the accuracy of prediction.

## Data Availability

The raw data supporting the conclusions of this article will be made available by the authors, without undue reservation.
